# Nano drug delivery systems: a promising approach to scar prevention and treatment

**DOI:** 10.1186/s12951-023-02037-4

**Published:** 2023-08-11

**Authors:** Jia-Ying Ding, Lu Sun, Zhi-Heng Zhu, Xi-Chen Wu, Xiao-Ling Xu, Yan-Wei Xiang

**Affiliations:** 1 Center of Rehabilitation Medicine, Yueyang Hospital, Shanghai University of Traditional Chinese Medicine, Shanghai, China; 2 School of Rehabilitation Science, Shanghai University of Traditional Chinese Medicine, Shanghai, 201203 China; 3 Shulan International Medical College, Zhejiang Shuren University, Hangzhou, 310015 PR China; 4 Department of Dermatology, Yueyang Hospital of Integrated Traditional Chinese and Western Medicine, Shanghai University of Traditional Chinese Medicine, Shanghai, China

**Keywords:** Scar formation, Nanomaterials, Prevention and treatment, Transdermal drug delivery

## Abstract

Scar formation is a common physiological process that occurs after injury, but in some cases, pathological scars can develop, leading to serious physiological and psychological effects. Unfortunately, there are currently no effective means to intervene in scar formation, and the structural features of scars and their unclear mechanisms make prevention and treatment even more challenging. However, the emergence of nanotechnology in drug delivery systems offers a promising avenue for the prevention and treatment of scars. Nanomaterials possess unique properties that make them well suited for addressing issues related to transdermal drug delivery, drug solubility, and controlled release. Herein, we summarize the recent progress made in the use of nanotechnology for the prevention and treatment of scars. We examine the mechanisms involved and the advantages offered by various types of nanomaterials. We also highlight the outstanding challenges and questions that need to be addressed to maximize the potential of nanotechnology in scar intervention. Overall, with further development, nanotechnology could significantly improve the prevention and treatment of pathological scars, providing a brighter outlook for those affected by this condition.

## Introduction

Skin serves as the primary barrier of the human body, protecting against harmful pathogens and chemicals, regulating temperature, and maintaining fluid balance [1]. Therefore, complete and healthy skin is crucial to the physiological health of the human body [2]. Scar formation is a normal physiological phenomenon that often occurs in the process of wound healing and manifests as the accumulation of extracellular matrix (ECM). However, it may cause an extremely heavy burden to patients and their families if it occurs after large-scale burns, operations, and trauma [3]. In general, normal skin tissues are viscoelastic, and the complete tissue structure confers certain tensile resistance to the skin, while the skin of scar tissue becomes harder and brittle, resulting in inferior biomechanical properties [4, 5]. Scar tissue, formed after skin injury, often lacks many functional structures, such as hair follicles, skin glands, dermal papilla, and other auxiliary components. This absence often leads directly to the permanent loss of the original function of the skin [6, 7]. Meanwhile, limitations of movement caused by wound contraction may lead to dysfunction [8].

Scar formation typically involves the inflammatory, proliferative, and remodeling phases [9]. The proliferative phase begins with the accumulation and proliferation of fibroblasts and is characterized by the formation of granulation tissue [10]. During this phase, fibroblasts form collagen to promote endothelial cell growth, while excessive proliferation of fibroblasts and deposition of collagen can lead to poorly structured fibrous tissue. The remodeling phase involves the degradation of type III collagen and replacement with type I collagen over one year [11]. Thus, preventing excessive fibroblast proliferation and collagen deposition and promoting well-structured fibrous tissue formation is crucial for effective scar prevention and treatment [12].

Scar formation is a complex biological process involving multiple factors. Of these, bacterial infection in skin wounds, inflammation, and disorganization of ECM are the major causes for the development of abnormal scars [13]. Skin flora or microbiota are associated with chronic wound healing following injury. When the skin is damaged, conditions become favorable for them to colonize and instigate infection within the skin, leading to undesirable clinical consequences. This process can produce various substances, such as reactive oxygen species (ROS), that cause oxidative damage and further kill the regenerating cells required for wound healing [14]. Meanwhile, persistent infections from flora or microbiota trigger an inflammatory cytokine storm, which further complicates wound healing [15]. Abnormal wound healing culminates in the formation of scars, including keloids and hypertrophic scars. Conversely, activated fibroblasts secrete excessive ECM, contributing to scar formation [16]. The abnormal accumulation and rearrangement of ECM promotes scar development.

**Fig. 1:**
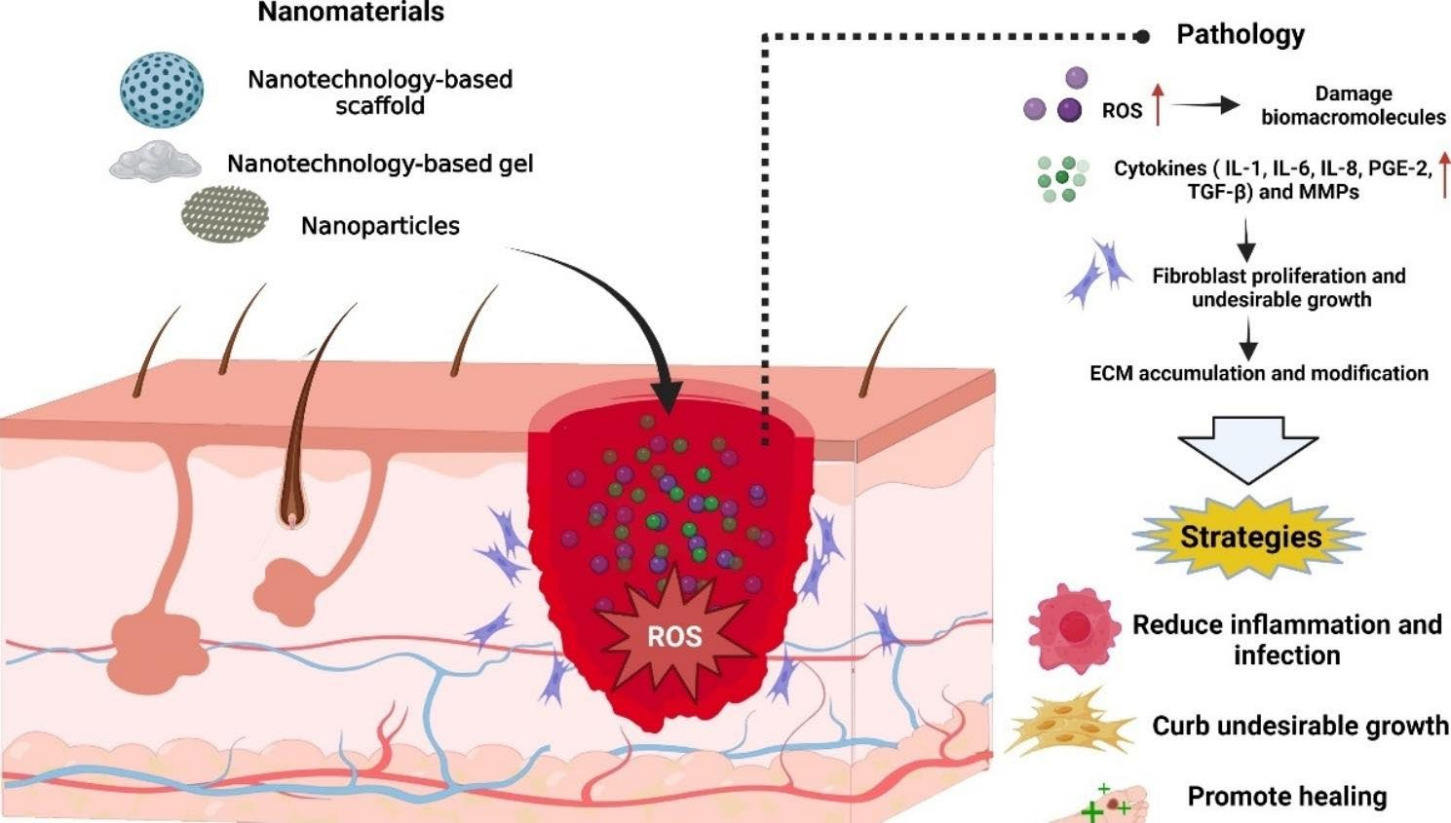
Strategies and nanomaterials to prevent and reduce scar formation

Our current understanding of scar formation mechanisms highlights three effective strategies to prevent and mitigate scar formation, as outlined in Fig. 1. The first strategy involves reducing inflammation and infection, which has been shown to be intimately related to scar formation. During the inflammatory stage, excessive reactive oxygen species (ROS) production can harm biomacromolecules, such as proteins, carbohydrates, and DNA. This damage spurs biochemical reactions leading to delayed healing and abnormal scar formation [17, 18]. Extended inflammation can stimulate the release of proinflammatory cytokines such as interleukin-6 (IL-6), interleukin-1 (IL-8), and matrix metalloproteinase (MMP) [19, 20]. In turn, MMPs can alter the extracellular matrix (ECM) and increase granulation tissue, driving the creation of excessive scarring [21, 22].

The second strategy is to curb undesired growth, particularly by attenuating the activity of cells contributing to scar tissue formation. Scar development can be inhibited by controlling key cytokines such as transforming growth factor-beta (TGF-β) and prostaglandin E2 (PGE-2). Reductions in TGF-β and increases in PGE-2 are associated with augmented collagen buildup and restricted collagen degradation [23, 24].

The third strategy emphasizes promoting healing. With a reduced risk of infection, fast healing posttrauma lessens the likelihood of scar formation [25]. Hence, numerous drugs targeting ROS and TGF-β can contribute to the prevention and reduction of scarring.

The formation of scars is a complex but self-limiting mechanism intrinsic to dermal wound healing. Despite the extensive studies conducted on wound healing, research on scar prevention and treatment remains woefully inadequate, necessitating further exploration. Presently, several interventions show promise in enhancing the aesthetics of scars, including compression therapy, laser treatment, radiotherapy, the use of silicone gel-formulated band-aids, and the topical application of corticosteroids via injections, tapes, plasters, and ointments [26, 27]. However, surgical procedures such as excision and skin grafting are reserved for scenarios where the scar encircles a joint, leading to significant dysfunction [28].

It is worth noting that these treatments, while effective, are not without their downsides, as they often lead to complications and adverse reactions. For example, laser treatments commonly induce transient erythema, a complication followed by the likelihood of blistering, purpura, edema, and further scarring [29]. Another case in point is compression therapy, which may give rise to tissue ischemia and diminished tissue metabolism [30]. Additionally, the usage of corticosteroids is linked to numerous negative effects that cannot be entirely circumvented [31].

As biotechnology evolves, the prospect of “scar-free healing” has emerged and generated significant interest. The underlying objective of this concept is to prevent scar formation entirely and instead foster tissue regeneration [32, 121]. It first took root in the study of fetal wound healing during gestation [33]. Research indicates that wounds heal quickly and without scarring in early- to mid-gestational mammalian fetuses [34]. In a promising progression, the concept of scarless healing has been introduced into the wound healing market in recent years. This was sparked by the discovery of target cells and signaling pathways, including profibrotic cell populations and the canonical Wnt/beta-Catenin signaling pathway. These discoveries aim to enable regenerative healing, and the early results have been promising [35].

While significant strides have been made in scar prevention and treatment, reaching beneath the skin barrier to the affected region remains a considerable challenge, primarily due to the excessive accumulation of collagen. The situation is further complicated by scar tissue lacking auxiliary features such as hair follicles and skin glands, making it even harder to penetrate [3]. On a positive note, nanomaterials have burst onto the scene, exhibiting great potential for scar treatment and prevention. Their application as a unique drug delivery strategy, especially in the domain of cancer research, is a promising development that has garnered substantial medical interest. The minute size of nanomaterials grants them an extensive specific surface area, enabling high drug loading capacity. Additionally, nanomaterials can be engineered to target precise locations for more efficient drug delivery. They can also solubilize hydrophobic drugs and extend drug release time. These remarkable properties position nanomaterials as significant tools in scar treatment.

This paper intends to consolidate and evaluate recent advancements in nanomaterial application for scar intervention and treatment in the past five years and analyze their merits and drawbacks. The findings from this study will provide valuable insights for the future deployment of nanotechnology in the realm of scar treatment.

## Nano drug delivery systems in scar intervention

The goal of scar intervention is to facilitate wound healing, prevent infection and inflammation, and inhibit adverse hyperplasia [36], and drugs are crucial in achieving these outcomes. However, drugs often face issues such as poor solubility, low stability, and rapid metabolism. The emergence of nanodrug delivery systems (NDDS) has helped address some of these issues. Therefore, we divided nanomaterials based on their composition into nanotechnology-based scaffolds, nanotechnology-based gels, and nanoparticles.

## Nanotechnology-based scaffolds

When a wound occurs, it is important to cover the wound site with a dressing that can prevent microbial invasion, maintain a moist environment, promote skin regeneration, and allow for gas exchange and exudate absorption [37]. Nanotechnology-based scaffolds is a good choice for their large surface area and high porosity, which are similar to the extracellular matrix of skin [38, 39]. These scaffolds not only provide suitable environment for wound healing, but also are excellent for drug delivery. Scaffolds, sponges, and membranes are common types of nanotechnology-based scaffolds that are often used as wound dressings [36]. Meanwhile, nanotechnology-based scaffolds also have the potential to inhibit undesirable growth due to their regular structure. Tables 1 and 2 summarize in vivo nanotechnology-based scaffolds in skin scarring and skin regeneration, respectively.

**Table 1:** In vivo Nanotechnology-based Scaffold in skin scarring

Nanomaterial	biomolecule or drug	Model	Major outcomes	Ref
Aligned carbon nanotubes (ACNTs) film	—	Rabbit ear model of hypertrophic scars (HS)	Ideal inhibitory effect on HS; suppressing cell proliferation, and guiding growth direction	[40]
Polycaprolactone(PCL) based electrospun nanofibrous mats (ENMs)	α-lactalbumin(ALA)	Rat deep second-degree burn model	Reduction of scar formation; accelerated wound healing and anti-inflammatory effects	[41]
Anisotropic Silver Nanoparticles (AgNPs) loaded Composite chitosan(Ch) electrospun nanofiber	Cur	Rat full-thickness skin wound model	Less scar formation; promotion of wound healing and antibacterial activity	[42]
Poly(3-hydroxybutyrate-co-3-hydroxyvalerate) (PHBV) nanofibrous meshes	—	Mouse full-thickness skin wound model	Mitigated excessive scar formation; improved re-epithelization and mitigated wound contraction	[43]
Cellulose acetate (CA)/soy protein hydrolysate (SPH) nanofibers scaffolds	—	Mouse excisional wound splinting model	Reduced scar formation; accelerated wound closure and tissue regeneration	[44]
Bilayer membranous (BLM) nanofiber scaffold	Decellular dermis matrix	Rabbit ear wound model	Inhibit the formation of hypertrophic scars; inhibit collagen fiber deposition and angiogenesis	[45]
Functionalized electrospun double-layer nanofibrous scaffold	Quaternized chitosan and silicone	Rabbit ear wound model	Inhibits scar formation, resists bacteria, promotes wound healing	[46]
Poly(ε-caprolactone)/gelatin (Gel) nanofibrous scaffolds (PCL/GE/PALs)	Palmatine	Rabbit ear model of HS	Significantly inhibition of HS formation; accelerated wound healing, decreased density of microvascular	[47]
Electrospun poly (L-lactide-co-glycolide)/gelatin (PLGA/Gel) membranes	ZnO nanoparticles and liraglutide	Rat bacterial-infected wound model	Scar length reduction; fast wound healing rate and antibacterial effect	[48]
Electrospun nanofibrous silk fibroin (SF)/GEL electrospun nanofiber	Astragaloside IV	Rat acute wound model	Anti-scar effect; accelerated healing, enhanced angiogenesis, and arrangement of collagen	[49]
Nanofibrous Electrospun Heart Decellularized Extracellular Matrix-based Hybrid Scaffold(NEhdHS)	—	Rat full-thickness skin wound model	Reduced scarring in the wound healing process	[50]
Rg3-loaded nanoin-micro electrospun composite fibers	20 S-Ginsenoside Rg3 (Rg3)	Rabbit ear model of HS	Inhibition of HS formation; reduced collagen deposition and vascularization; more sustainable drug release	[51]
PLGA nanoparticles in polyethylene glycol diacrylate (PEGDA) core/alginate shell structured hydrogel particles	hydrophobic corticosteroid	Rabbit ear wound model	Exhibited suppress scar formation; sustainable drug release over 4 weeks	[52]
Cerium oxide (CeO_2_) nanocapsules (NCs) Adhered plasma-etched polylactic acid (PLA)-fiber membranes	Pirfenidone(PFD)	Mouse wound-healing odel	Satisfactory wound-repairing and anti-scarring effects	[53]
Cerium oxide nanoparticles (CONPs)-loaded Poly-L-lactic acid (PLLA)-gelatin composite fiber membranes	—	Rats scalding model	Better scar remodeling effect and regenerative performance compared to other groups	[54]

**Table 2:** In vivo Nanotechnology-based Scaffold in skin regeneration

Nanomaterial	biomolecule or drug	Model	Major outcomes	Ref
Nanofibrous scaffolds comprising polyvinyl pyrrolidone (PVP), cerium nitrate hexahydrate (Ce(NO_3_)_3_·6H_2_O)	Curcumin(Cur)	Rat full-thickness skin wound model	Scarless wound healing; reduced local oxidative stress	[55]
Dextran based bionanocomposite membranes	Clove oil (CO) and sandalwood oil (SO)	Mouse full-thickness skin wound model	Scarless wound healing; proper collagen formation and organization, and presence of hair follicles	[56]
Sulphonated polyether ether ketone (SPEEK) nanofibrous scaffold	Aloe vera	Rat excision skin wound model	Scarless wound healing; excellent pathogenic inhibition	[57]
Amniotic membrane (AM)/SF membrane	Adipose tissue-derived mesenchymal stem cells	Mouse third degree burn wound model	Scarless wound healing; accelerated wound healing, neo-vascularization and early re-epithelialization	[58]
Ag/Glass–ceramics (GC)-Ch/polyethylene oxide (PEO)/Gel electrospun scaffolds.	Mouse embryonic fibroblasts	Mouse full-thickness skin wound model	Scarless cutaneous wound regeneration; enhanced angiogenesis, and collagen synthesis as well as regeneration of the sebaceous glands and hair follicles	[59]
Silver-catechin nanocomposite tethered collagen scaffolds	Silver-catechin nanocomposite	Rat full-thickness skin wound model	Scarless wound healing; increased angiogenesis	[60]
ZnO–curcumin nanocomposite tethered collagen scaffolds	ZnO–curcumin nanocomposite	Rat burn wound healing model	Scarless wound healing; upregulates angiogenesis and TGF-β3 expression thereby	[61]

One important form of nanotechnological application in wound care is the use of nanofibrous scaffolds. PLA, PLLA, PCL and PLGA are the most frequently used biodegradable materials for biomedical applications [62], and common material for nanofibrous scaffold. Nanofibrous scaffolds have a high surface-volume ratio, small aperture, and adjustable and flexible function, as well as being porous enough to allow for cell infiltration and intercellular interactions [63–65]. They can create an artificial environment that resembles natural tissue, providing greater mechanical support for cell attachment and migration [66].

Moreover, silk fibroin (SF) is a kind of natural polymer fibroin extracted from silk with good biocompatibility and partial biodegradability. It has no skin irritation, no toxic side effects, and has sustained release performance, gas permeability and moisture permeability [67]. However, the degradation of silk fibrin is very slow. To solve this problem, researchers added gelatin to develop AS-loaded SF/GT nanofiber dressing, which shows accelerated healing and excellent anti-scaring effect [49].

Permutation structure materials have the potential to promote the arrangement of wound fibers and enhance tissue regeneration. Research has shown that the orderly alignment of polymeric biomaterials can induce cell arrangement and affect the structure of the ECM, thereby influencing keratinocyte behavior [68, 69]. However, the methods of fabricating nanofibrous scaffolds are limited, most nanofibrous scaffolds are fabricated by electrospinning technique, which means mass production is difficult [70]. ACNT, as a kind of inorganic nanomaterial, comes into being. The most obvious advantages of ACNT are acceptable cost and potential for scale production. Researchers found that it has an arranged structure with appropriate fiber diameter and spacing, which curbs undesirable growth by inhibiting the TGF-β pathway, with an impressive effect on preventing HS [40].

Traditional nanofiber materials like Poly-3-hydroxybutyrate (PHB) and PHBV possess large stiffness and high levels of endotoxin for which have hindered their use. To address this issue, researchers have developed methods for synthesizing PHBV polymers using haloarchaea as a cell factory, resulting in significantly lower endotoxin levels compared to those produced by bacteria [43]. In a study using a full-thickness wound mouse model, these PHBV nanofiber scaffolds effectively mitigated excessive scar formation, demonstrating their potential for use in tissue engineering applications [71].

Multifunctional composite scaffolds are superior to single-material scaffolds because they can simulate the structure of ECM, creating a more suitable microenvironment for tissue regeneration. ALA is a dietary protein that is rich in tryptophan, which acts as a precursor to the neurotransmitter serotonin. ALA has the potential to promote burn wound healing and reduce scarring. By adding ALA to PCL using different mass ratios and electrospinning the mixture, ALA/PCL-based ENMs can be fabricated. These ENMs are more similar in structure to natural ECM than traditional scaffolds [41], and can promote the synthesis of type I collagen and increase the ratio of type I collagen to type III collagen, which improves collagen maturation [72]. Soy protein is another material that is similar in bioactive molecules to ECM proteins and estrogen. SPH nanofiber scaffolds are developed using rotary jet spinning, and this scaffold successfully simulates the physicochemical properties of natural skin ECM. It exhibits a high water retention ability, ultimately reducing scar formation and collagen anisotropy [43].

Recently, there has been a growing interest in the use of three-dimensional (3D) printing to help develop nanofiber scaffolds that resemble skin structures. One such scaffold is BLM nanofiber scaffold, which uses 3D printing technology to prepare double-layer scaffold first, and then prints dECM solution on the surface of the prepared PLGA membrane [44]. BLM stents have been shown to inhibit the deposition of collagen fibers and angiogenesis, thereby preventing the formation of hyperplastic scars. The use of 3D printing technology means that there is a broader application prospect for nano scaffolds.

In addition to these, nanotechnology-based scaffolds have also been bestowed with antibacterial properties. Essential oils (EOs), such as lemongrass, cinnamon, and mint, have also been studied for their antibacterial activity with low toxicity and high biocompatibility [73, 74]. CO and SO encapsulated in dextran-based nanocomposite membranes have been found to be effective in preventing microbial invasion [56]. This modification of the wound healing cascade results in complete healing within 14 days, indicating a significant effect on scar prevention.

**Fig. 2:**
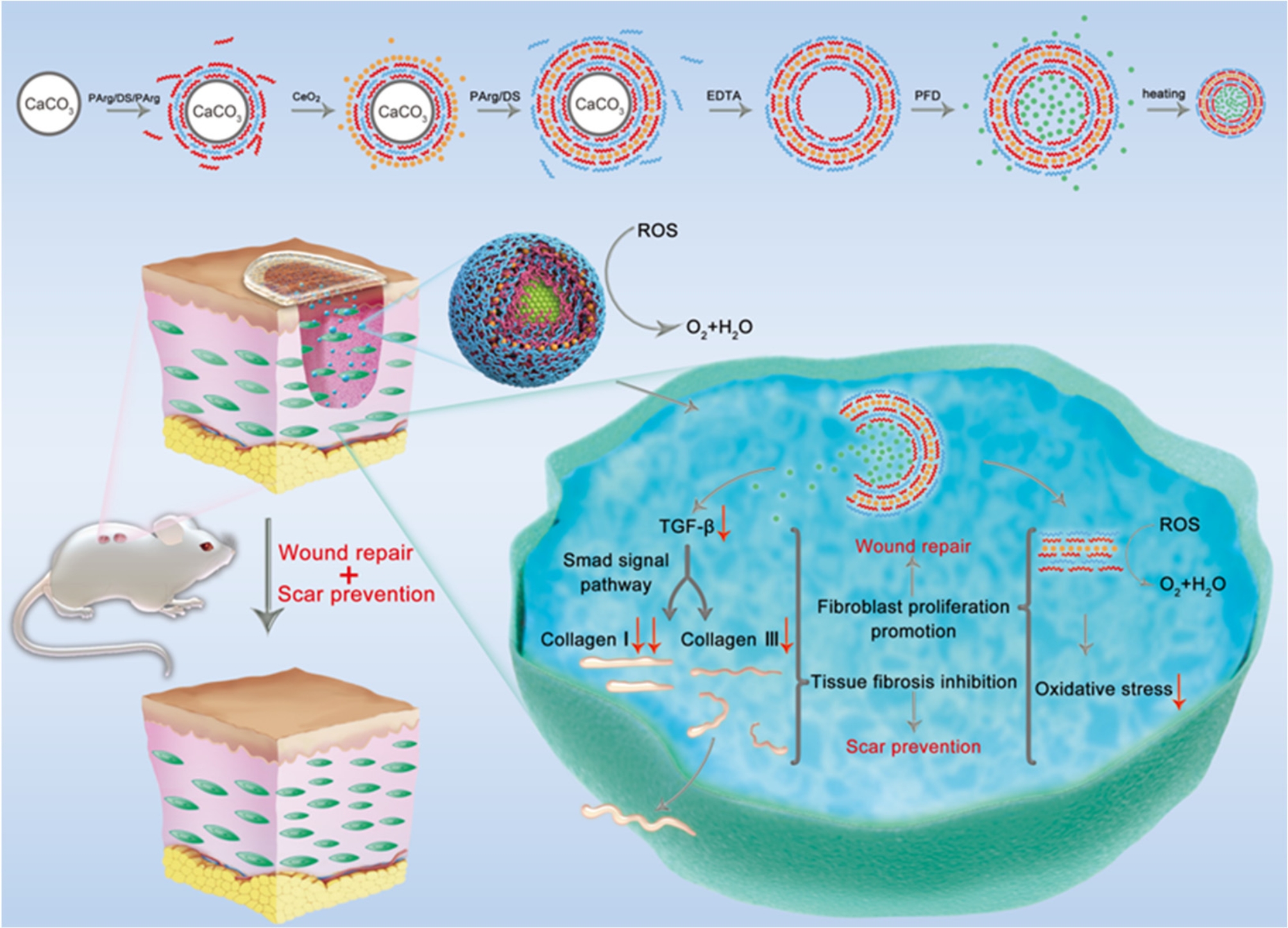
Through one-step precipitation, Rg3 was highly effectively loaded in nanoparticles (PN/Rg3), and finally, through nano-micro strategy technology, electrospun fibers loaded with PN/Rg3 were successfully engineered (P-PN-Rg3 fibers). Adapted, with permission

Nanotechnology-based scaffolds are also suitable for the delivery. CeO2 is a type of ROS scavenger, but it is easily adsorbed by biomacromolecules, which reduces its enzyme-like activity. To address this limitation, He et al. [53] designed NCs with a sophisticated structure carrying PFD and adhering to PLA fiber film using layer-by-layer methods. The resulting scaffold demonstrated significant wound repair and an anti-scar effect. To address the issue of poorly soluble drug delivery, two-stage loading has been used to prolong the release of the loaded drug, as shown in Fig. 2. This involves using a poly(N-isopropyl acrylamide) nanoparticle with reversible hydrophilic-to-hydrophobic properties as a drug delivery carrier that is introduced into electronspun fibrous as a medical scaffold. The results of this approach suggest that the nano-micro scaffold is effective in inhibiting scarring [51].

## Nanotechnology-based gel

To put it simply, nanotechnology-based gel refers to the introduction of nanoparticles or nanostructures into the molecular network of gels through physical or chemical crosslinking methods [75]. This has become a popular area of research in the past decade due to the rapid development of nanotechnology and its potential for drug delivery. Tables 3 and 4 summarize the in vivo nanotechnology-based gel in skin scarring and skin regeneration, respectively.

**Table 3:** In vivo Nanotechnology-based gel in skin scarring

Nanomaterial	biomolecule or drug	Model	Major outcomes	Ref
In situ gel composed of self-assembled lattice nanostructures	PFD	Mouse deep partial thickness (DPT) burn	Scar inhibition: accelerated healing process and shortened inflammation phase	[76]
Carboxymethyl chitosan (CMC)/aldehyde-modified CNC (DACNC) nanocomposite self-healing hydrogels	—	Rat deep partial thickness burn model	Prevent scarring; accelerated deep partial thickness burn wound healing	[77]
Nanoethosome Gels	ALA	Rabbit HS models	Improved HS; remodeling collagen fibers	[78]
Gelatin methacryloyl-dopamine(GelMA-DOPA) hydrogel	CONPs and an antimicrobial peptide (AMP)	Rat wound and infection model	Decreased scar formation; accelerated wound healing.	[79]
AgNPs gels	—	Methicillin-Resistant Staphylococcus pseudintermedius(MRSP) infected mice wound model	Reduced scar appearance; improved collagen fiber alignment and reduced pus formation	[80]
Fe-SiO2 nano composites membrane and hydrogel	Curcumin	Mouse full-thickness skin wound model	Inhibiting scar hyperplasia; promoting hair follicle regeneration.	[81]
Nanoethosomes gel	IR-808	Rabbit ear HS model	Remarkable therapeutic effects on improving the HS appearance, promoting HSF apoptosis and remodeling collagen fibers	[82]
Self-assembled peptide-hydrogels	Resveratrol	Rat full-thickness skin wound model	Scar inhibition; Accelerated wound healing, well-organized collagen deposition, reduced inflammation	[83]
Composite hydrogel is composed of modified polycaprolactone nanofiber with plasma treatment	—	Mouse full-thickness skin wound model	No obvious scar; promotion of skin wound healing	[84]

**Table 4:** In vivo Nanotechnology-based gel in skin regeneration

Nanomaterial	biomolecule or drug	Model	Major outcomes	Ref
Sodium alginate gum acacia polymeric nanocomposite hydrogels	Zinc oxide nanoparticles (ZnO NPs)	Rabbit full-thickness excision model	Scarless wound healing; reduced inflammation, accelerated healing,	[85]
Intercalated N-CNS polymer nanocomposites (ICPN) and HCPN (heptazine N-CNS polymer nanocomposites)	Losartan	Rat deep second-degree wound model	Excellent scarless healing	[86]
(PAGE)-based nanogel	CONPs and curcumin	Rat full-thickness skin wound model	Scarless healing; early resolution of inflammation and regrowth of hair follicles	[87]
MXene nanofibers @ VEGF core with dopamine-hyaluronic acid hydrogel@dopamine shell(MNFs@V–H@DA)	VEGF	Mouse wound-healing model	Scarless healing; appropriate vascularization, anti-inflammatory effects	[88]
AgNPs/Glucose oxidase nanocapsules (nGOx)/apramycin (Apra) nanocomposite gel	Glucose oxidase (GOx)	Rabbit coli-induced inflammation model and mice Propionibacterium acnes-induced inflammation model	Rapid scarless skin recovery; significant bacterial growth inhibition and broad antimicrobial spectra	[89]
Silk nanofiber hydrogels	Bone marrow mesenchymal stem cells (BMSCs)	Rat full-thickness skin wound model	Scarless healing with hair follicle recovery	[90]
silk nanofiber hydrogels	Asiaticoside(AC)	Rat full-thickness skin wound model	Scarless wound repair; regulated inflammatory reactions and angiogenesis	[91]

Hydrogels themselves are known for their strong hydrophilicity and biocompatibility, making them ideal for medical applications. According to the “wet wound healing theory”, wounds heal twice as fast in a wet environment as they do in a dry environment [92]. The combination of nanomaterials and hydrogels can further enable functions such as exogenous stimulus response to achieve more precise drug release. Nanoparticles have unique physical and chemical properties that, when combined with hydrogels, can enhance their mechanical properties, and facilitate controlled drug release.

Different gel materials have different properties in preventing scars. A nanocomposite self-healing hydrogel is developed that can be injected into irregular and deep burn wound, followed by rapid self-healing into a complete hydrogel, thoroughly filling the wound area and protecting the wound site from the external environment. The hydrogel is made from naturally occurring polymers, CMC and DACNC. And finally it dissolves as needed and take advantages of amino acid solution to accelerate the healing of deep burn wound and prevent scarring [77].

Inappropriate wound healing and scar formation can be caused by two major problems in wound tissue: elevated levels of ROS and high expression levels of TGF-β [44]. Inflammatory immune cell bursts can lead to the overproduction of ROS, which can also damage biomacromolecules [93]. However, delivering anti-ROS drugs and agents can be challenging. Hence, using an effective enzyme-like ROS scavenger can be an effective strategy for managing ROS levels. PFD is an antifibrotic agent that can inhibit the expression of TGF-β, making it a promising treatment for scars. However, due to its small molecular weight and instability to ROS, efficient cellular delivery of PFD can be challenging [94]. To address this issue, a hyaluronic acid combined lyotropic liquid crystal-based spray dressing (HLCSD) loaded with PFD has been developed for managing DPT burn wounds [76]. The material has a low viscosity liquid form before administration, making it easy to apply as a spray and ensuring even wound coverage. Once in contact with the wound, water triggers a unique phase transition from the spray precursor to a gel. The PFD-loaded HLCSD has demonstrated effectiveness in promoting healing and preventing scars, showing significant promise as a spray dressing for DPT burn injuries.

To prevent infection and promote wound healing, antibacterial ingredients are often added to wound dressings. Silver ions are a popular additive for skin tissue engineering scaffolds due to their antibacterial properties. When combined with nGOx and Apra, the nanocomposite gel matrix successfully inhibit bacteria [89]. Inspired by mussels, researchers prepared a hydrogel dressing with improved binding affinity to wet skin surfaces by the combination of GelMA with DOPA and adding AMP. The results show thatAMP released from the hydrogel has strong antibacterial activity and has obvious effects on the four representative strains [79].

In addition, nanotechnology-based gel can achieve targeted delivery and controlled release of drugs. In recent years, photodynamic therapy (PDT) has gained significant attention as a new therapeutic method, particularly for the treatment of tumors. In this therapy, nanomaterials with photodynamic ability are irradiated to produce ROS, which can effectively destroy and eliminate tumor cells. 5-Aminolevulinic acid (ALA) is a second-generation photosensitizer that is commonly used in PDT and has the potential to target PDT for hypertrophic scars. However, the main challenge with its application is how to penetrate the skin barrier and stay at the scar site for an extended period. To address this challenge, nanoethosome gels (EGs) are applied as delivery carriers [78]. The experimental results demonstrated that EGs could effectively pass through the scar model, exert a photothermal effect, and inhibit hyperplasia of the scar.

Nanofiber and hydrogel materials each have unique advantages that make them useful in wound dressing applications. Nanofibers are often used to meet mechanical requirements, while hydrogels are better at exchanging with cells or tissues due to their high water content. However, fiber materials used as drug carriers can be limited by fiber interface dilatation affecting drug release. Hydrogels, on the other hand, can regulate exchange rates with cells or tissues through the microenvironment. Therefore, combining nanofibers and hydrogels as carriers can be an excellent wound dressing material that allows for accurate drug release [90]. For example, a hydrophobic drug, AC, was loaded inside silk nanofiber hydrogels to create bioactive and injectable matrices for skin regeneration. AC retains its biological functions of regulating the inflammatory response and vascularization by dispersing in water-based silk nanofiber hydrogels, achieving scarless wound repair [91].

## Nanoparticles

In addition to nanotechnology-based hydrogels and scaffolds, some nanoparticles are applied to the wound site in solution to prevent scarring. On the one hand, any wound dressings of hydrogels and scaffolds play a role in the middle and late stages. However, for diabetic patients, hyperglycemia induces excessive production of ROS through the mitochondrial electron transport chain and advanced glycation end products, leading to sustained oxidative stress and inflammation in diabetic wounds [95, 96]. Hence, early interventions to wound are warranted. On the other hand, the solution with Nanoparticles in contact with the wound can exert a sustained release effect. Tables 5 and 6 summarize in vivo nanoparticles in skin scarring.

**Table 5:** In vivo nanoparticles in skin scarring

Nanomaterial	biomolecule or drug	Model	Major outcomes	Ref
CONPs	—	Rabbit ear scar model	Improved the scar appearance and collagen arrangement	[3]
Liposome	Statins	Rabbit ear models of HS	Significantly reduced hypertrophic scarring; reduced erythema/vascularity of scars	[97]
DNA-Fe nanoparticles	Doxorubicin hydrochloride (DOX)	Rabbit HS models	Scar-inhibiting effects; penetration ability, rapid drug release	[98]
Light-controlled thermosensitive Nanoformulation(TSLC)	Cell membrane repair protein (rhMG53)	Rat diabetic model with full-thickness cutaneous wounds	Reduction in scar formation; inhibited excessive skin fibrosis, angiogenesis, and increased wound closure rate	[99]
Papain elastic liposomes (PEL)	Papain elastic	Rabbit ear model	Improved HS; significantly decreased microvascular density, and collagen fiber	[100]
Selenium@SiO_2_ nanoparticles (Se@SiO2NPs)	—	Rat full-thickness skin wound model	Suppressed the formation of hypertrophic scars and accelerated dermal wound healing, accompanied by oxidative stress inhibition	[101]

**Fig. 3:**
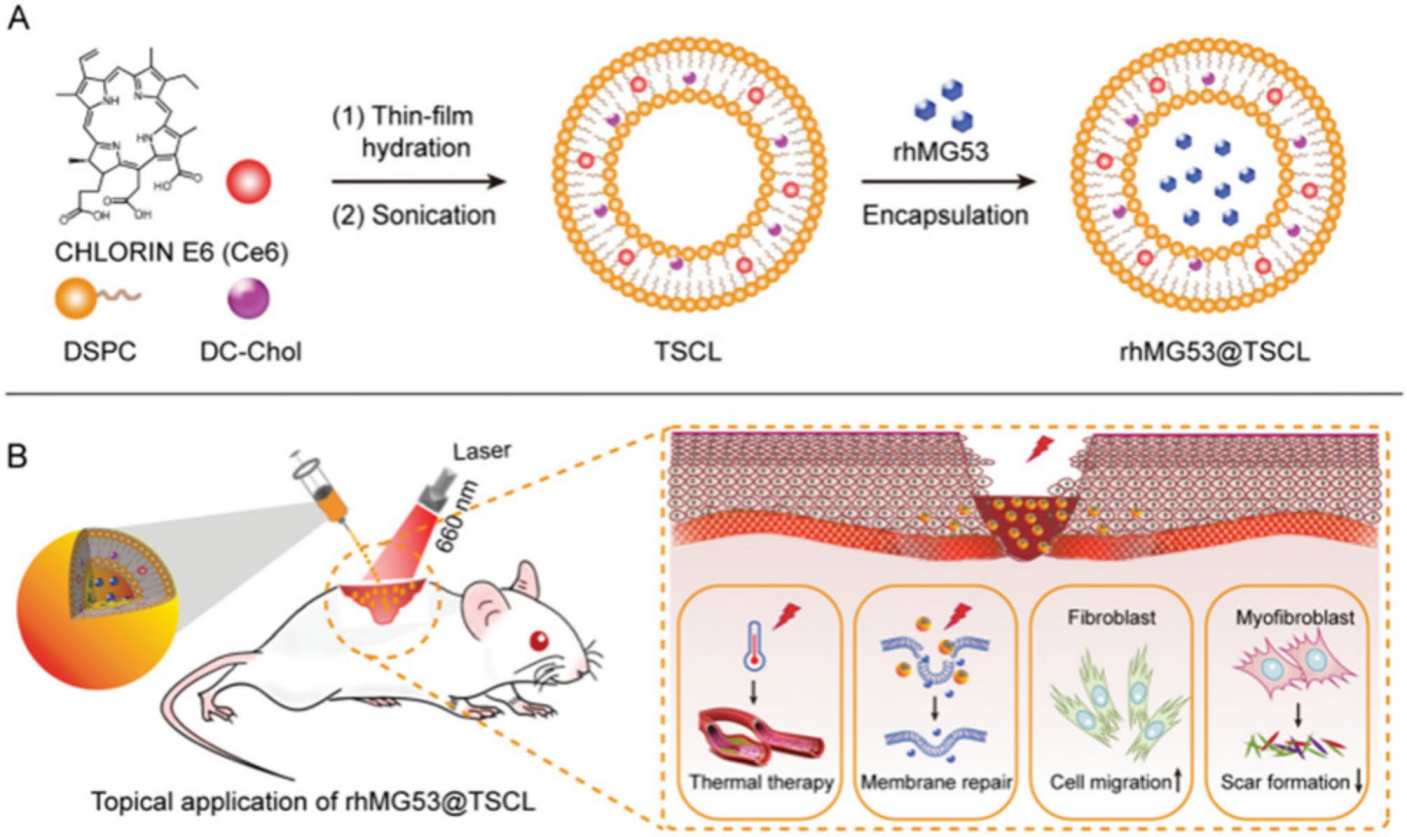
(**A**) Composition and self-assembly process of rhMG53@TSLC. (**B**) Scheme and synergistic treatment mechanisms of photothermal therapy for scarless wound healing. Adapted, with permission

Damage to cell membrane repair plays a crucial role in the occurrence and development of inflammation in the early stage of diabetic wound formation. However, rhMG53, which plays a key role in the repair of cell membrane damage, is easily degraded in tissues [102]. To address this issue, a remote light-controlled thermosensitive nanoformulation was developed. This formulation integrates the photothermal conversion properties of a photosensitizer and rhMG53 (Fig. 3). Under photothermal stimulation, the nanoformulation can protect and effectively release rhMG53 to control cell membrane damage at an early stage, thereby inhibiting excessive skin fibrosis and angiogenesis and promoting scar-free healing [99].

It is well known that ROS can cause the destruction of cell membranes, which can be detrimental to the wound healing process. To maintain the benefits of scavenging ROS, it is essential to develop a drug delivery system with a slow-release effect. To achieve the purpose of sustained release, Selenium, which is a common component of antioxidant drugs through glutathione peroxidase [103], also capsuled into the nanoparticles. Se@SiO_2_NPs shows excellent slow-release ability, and biosafety and can promote scarless wound healing [101].

Fibroblasts are the most important effector cells associated with scar formation, and they are typically activated in inflammatory responses and closely related to scar hyperplasia. Therefore, inhibiting the growth of fibroblasts is an essential strategy for inhibiting scar formation. In recent years, nanoparticles have shown great potential in inducing endogenous and extrinsic apoptotic pathways. CONPs have been widely used in many antitumor therapies in recent years due to their extensive antitumor properties, which include inhibiting tumor cell proliferation and inducing tumor cell apoptosis. To test the effectiveness of CONPs in inhibiting fibroblast growth and promoting apoptosis, an intralesional injection of CONPs infused with cuprous oxide was applied in vivo [3]. This approach has shown therapeutic potential for treating hypertrophic scars.

## Discussion

Skin scarring refers to fibrous tissue that is not completely remodeled by skin injury. It can cause stiffness, loss of function, and poor appearance, leading to a variety of physiological and psychological problems. This can result in a heavy burden for both patients and the healthcare system. Despite the serious situation, there is still a lack of effective strategies for preventing scar formation, let alone methods for remodeling mature scars [104]. Traditional methods for preventing scar formation include incisions along the Langers line, special deep sutures, and dressings to reduce wound tension [105]. The prevention and treatment of scars are challenging.

The adverse effects of infection on wounds are indisputable. Nanomaterials have significant advantages in anti-infection. First, wound dressings based on nanomaterials can meet the needs of gas exchange due to their high porosity and can also act as a microbial barrier. It can adsorb excess exudates, inhibit microbial growth, and promote healing [106]. In addition, the introduction of some antibacterial components, such as silver nanoparticles, can kill harmful bacteria and keep the wound in a suitable environment for healing.

Despite numerous methods used to reduce scars, the insignificant therapeutic effect and numerous side effects make the achievement of scarless wounds a significant challenge. The limited efficacy of topical plaster and ointment, the risk of infection due to injection and surgery, and adverse reactions caused by treatment, such as pain, permanent hypopigmentation, and skin atrophy, make drug delivery for scars a challenging endeavor [107]. Traditional wound dressings, such as gauze, can only temporarily cover and protect the wound. However, these dressings do not effectively control the humidity of the wound microenvironment and can cause secondary damage by generating adhesion to the wound site leading. Additionally, it is difficult to prevent and inhibit the invasion of pathogenic microorganisms during long-term use, which increases the risk of wound infection.

Scars often result in cuticle barrier dysfunction due to the absence of skin glandular structures such as hair follicles and skin glands and the lack of mature cuticle cells [108, 109]. Excessive proliferation of fibroblasts and excess collagen deposition are often involved in scar maturation [110]. The abnormal structure of these scar tissues creates a biological barrier that limits drug penetration to the fibroblasts in the deep scar tissue [111]. This makes it difficult to achieve effective drug delivery to scar tissues, and fluid dilution, enzyme degradation, and natural obstacles are the main obstacles that limit drug retention time and efficacy. Routine administration is often associated with systemic adverse reactions due to nonspecific biological distribution and uncontrolled release [112].

In addition to adverse reactions, drugs used for scar treatment also face other challenges, such as poor solubility, low stability, and fast metabolism, which can affect their efficacy. Furthermore, these drugs often lack targeting functions and may have potential toxic and side effects. Scar formation also involves the abnormal alignment of collagen fibers, which is difficult to address with traditional drugs, making scar-free healing even more challenging.

Preventing and treating scars while minimizing toxic side effects is a great challenge. However, percutaneous administration and nanotechnology-based materials have shown promise in delivering therapeutic drugs into the body safely and effectively. Nanotechnology-based materials have good biocompatibility and can penetrate biological barriers easily, which can solve the problem of deep penetration of drugs. They can also be absorbed and utilized by tissues, thus stimulating tissue regeneration [113]. Nanocarriers can protect drugs and release them slowly, improving the blood circulation time of drugs in the body. Additionally, the response ability of the nanodrug delivery system can be adjusted to various stimuli, such as magnetic fields, light, electric fields, ROS and other exogenous stimuli, to achieve accurate drug delivery location and reduce the toxicity and side effects of drugs [114]. In addition, NDDSs have high drug loading efficiency and can deliver two or more drugs at the same time, inhibiting microbial infection and promoting tissue regeneration. What’s more, nano scaffolds provide a high surface area for growth, promoting wound healing, rapid cell proliferation, and granulation. Furthermore, they can induce the orderly arrangement of collagen fibers, which has significant potential to promote wound healing and skin repair.

Scarless wound healing is still the goal to be pursued. According to the existing research, combining one or more nanomaterials can be more effective in combating scars. If the nanotechnology-based scaffold is combined with gel, the advantages of both are reflected, such as forming a wound dressing more like ECM. In addition, the fusion of multiple anti-scar strategies was also associated with better scar healing. A combination of strategies to promote healing and inhibit undesirable growth is also commonly used and has been shown to bring more significant anti-scarring effects. Based on this idea, considering appropriate anti-scar strategies based on their mechanisms when designing nanomaterials may bring better results.

Despite the potential benefits of NDDS for scar treatment, there are still challenges that need to be addressed. First, is the biological safety of nanomaterials. Biomedical use of nanomaterials requires low or no toxicity. Generally, the cytotoxicity of nanomaterials is related to the surface properties, size and in vivo distribution of nanoparticles. Nanomaterials are associated with more pronounced toxic reactions due to their small size [115]. Nanoparticles smaller than approximately 10 nm in diameter may affect the kidney, while nanoparticles larger than 200 nm may activate the complement system [116]. At the same time, the distribution of nanomaterials in the body is very important. Skin is a relatively safe application site, and the nanomaterials applied to skin are generally nontoxic and low toxic by in vitro experiments and have less impact on wounds. For example, ACNTs have been proven to be highly biocompatible and nontoxic by in vitro toxicity experiments, but relevant toxicity studies in vivo are lacking [40]. Therefore, whether the nanomaterials applied to the wound site will enter the blood circulation and reach other parts of the body after contact with the skin is a question worth considering.

Moreover, large-scale clinical studies are needed to demonstrate their effectiveness in scar treatment. In terms of scar treatment, research on the molecular mechanism of fibrosis has produced several new targets, such as TGF-β [117]. However, these targets have been tested clinically, and the results are not optimistic. The biodegradability and cytotoxicity of some nanomaterials still need further investigation. For example, some nanofiber scaffolds have poor biocompatibility in vivo, and a considerable amount of research can improve their biocompatibility by fusing nanofibers with gels. In addition, the toxicity of drugs can also be solved by enhancing targeting. At present, most nanodelivery systems for scar intervention are mainly passive targeting, so active targeting with a stimulus response can be considered. For example, the delivery vector of 5-aminolevulinic acid adopts a photothermal response. It allows the drug to be released at the target site to reduce the impact on other sites to achieve precision therapy.

The healing of large wounds requires skin substitutes to reduce wound contracture, realizing scar-free healing and improving skin regeneration. The skin has a multilayered structure, and a composition similar to that of the skin is more conducive to wound healing. However, the current methods of making nanofiber scaffolds have a single composition and structure, and it is difficult to achieve many functions. Three-dimensional printing nanocomposites have emerged as a solution to overcome the deficiencies associated with these nanomaterials. For example, a novel skin substitute was developed by combining a multilayer skin tissue reconstruction method with nanofibers and microfibers loaded with human keratin extract using electrospinning and support structures using 3D printing. Keratinocytes and fibroblasts form epidermis and dermis on PCL/keratin scaffolds, respectively, and realize skin regeneration [118]. Wu et al. the Silver-Ethylene Interaction and 3D Printing develop Antibacterial Superporous Hydrogels. They used 3D printed templates and HPMC as the pore-making materials. Printed templates and HPMC as the pore-making materials resulted in regular large pores. Promote the healing of infected wounds and inhibit the formation of scar tissue [119]. Moreover, a dual piezoelectric response model that can be used to simulate and amplify endogenous bioelectricity can also be produced by 3D printing. The researchers used zinc oxide nanoparticles to improve the hydrophilicity of PVDF and polarized 3D printing piezoelectricity, two hydrogel materials with opposite wetting behaviors, and added sodium alginate (SA) to make PVDF easy to cross link through Ca2+. Finally, a porous mesh structure with a dual piezoelectric response model was fabricated to significantly improve skin regeneration [120].

To date, there have been few reports on the clinical application of electrospun nanofiber membranes as wound dressings. This may be due to the complex production processes involved in many nanomaterials used for drug delivery, as well as strict equipment requirements, which make mass production difficult to achieve. Meanwhile, diverse parameters affecting the morphology, complex structure of nanomaterials, and uncontrollable electrospinning process make it difficult to obtain nanofibers accurately and stably. The current challenge is to scale up the production of these devices and achieve clinical translation of these technologies. One possible solution is to combine 3D printing technology to prepare nanomaterials, which may help to optimize the production process to some extent. However, there are still many technical challenges to be addressed before 3D printing of biological materials can be successfully translated from research to clinical use. Another potential approach is to learn from the improvements made in the production efficiency of ACNTs and make further efforts to improve the efficiency of nanomaterial production.

Despite the challenges, it is clear that nanomedicine has great potential and has already seen significant advances. However, there is still a long way to go before these technologies can be widely adopted in clinical practice.

## References

[1] Lin X, Li YZ, Chen T, Min SH, Wang DF, Ding MM, Jiang G (2022). Effects of wearing personal protective equipment during COVID-19 pandemic on composition and diversity of skin bacteria and fungi of medical workers. J Eur Acad Dermatol Venereol.

[2] Tenno M, Shiroguchi K, Muroi S, Kawakami E, Koseki K, Kryukov K, Imanishi T, Ginhoux F, Taniuchi I (2017). Cbfβ2 deficiency preserves Langerhans cell precursors by lack of selective TGFβ receptor signaling. J Exp Med.

[3] Xiao Y, Xu D, Song H, Shu F, Wei P, Yang X, Zhong C, Wang X, Müller WE, Zheng Y, Xiao S, Xia Z (2019). Cuprous oxide nanoparticles reduces hypertrophic scarring by inducing fibroblast apoptosis. Int J Nanomedicine.

[4] Fernandes MG, da Silva LP, Cerqueira MT, Ibañez R, Murphy CM, Reis RL, Brien O, Marques FJ (2022). Mechanomodulatory biomaterials prospects in scar prevention and treatment. Acta Biomater.

[5] Dunn MG, Silver FH, Swann DA (1985). Mechanical analysis of hypertrophic scar tissue: structural basis for apparent increased rigidity. J Invest Dermatol.

[6] Tredget EE, Nedelec B, Scott PG, Ghahary A (1997). Hypertrophic scars, keloids, and contractures. The cellular and molecular basis for therapy. Surg Clin North Am.

[7] Castleberry SA, Golberg A, Sharkh MA, Khan S, Almquist BD, Austen WG, Yarmush ML, Hammond PT (2016). Nanolayered siRNA delivery platforms for local silencing of CTGF reduce cutaneous scar contraction in third-degree burns. Biomaterials.

[8] van Zuijlen PP, Ruurda JJ, van Veen HA, van Marle J, van Trier AJ, Groenevelt F, Kreis RW, Middelkoop E (2003). Collagen morphology in human skin and scar tissue: no adaptations in response to mechanical loading at joints. Burns.

[9] Greaves NS, Ashcroft KJ, Baguneid M, Bayat A (2013). Current understanding of molecular and cellular mechanisms in fibroplasia and angiogenesis during acute wound healing. J Dermatol Sci.

[10] Singer AJ, Clark RA (1999). Cutaneous wound healing. N Engl J Med.

[11] Profyris C, Tziotzios C, Do Vale I (2012). Cutaneous scarring: pathophysiology, molecular mechanisms, and scar reduction therapeutics part I. The molecular basis of scar formation. J Am Acad Dermatol.

[12] Shirakami E, Yamakawa S, Hayashida K (2020). Strategies to prevent hypertrophic scar formation: a review of therapeutic interventions based on molecular evidence. Burns Trauma.

[13] Zhang C, Yang D, Wang TB, Nie X, Chen G, Wang LH, You YZ, Wang Q (2022). Biodegradable hydrogels with photodynamic antibacterial activity promote wound healing and mitigate scar formation. Biomater Sci.

[14] Haq A, Kumar S, Mao Y, Berthiaume F, Michniak-Kohn B (2020). Thymoquinone-loaded polymeric Films and Hydrogels for Bacterial Disinfection and Wound Healing. Biomedicines.

[15] Huang D, Song SJ, Wu ZZ, Wu W, Cui XY, Chen JN, Zeng MS, Su SC (2017). Epstein-Barr Virus-Induced VEGF and GM-CSF Drive Nasopharyngeal Carcinoma Metastasis via Recruitment and activation of macrophages. Cancer Res.

[16] Jones EM, Cochrane CA, Percival SL (2015). The Effect of pH on the Extracellular Matrix and Biofilms. Adv Wound Care (New Rochelle).

[17] Wu H, Li F, Wang S, Lu J, Li J, Du Y, Sun X, Chen X, Gao J, Ling D (2018). Ceria nanocrystals decorated mesoporous silica nanoparticle-based ROS-scavenging tissue adhesive for highly efficient regenerative wound healing. Biomaterials.

[18] Giroux X, Su WL, Bredeche MF, Matic I (2017). Maladaptive DNA repair is the ultimate contributor to the death of trimethoprim-treated cells under aerobic and anaerobic conditions. Proc Natl Acad Sci U S A.

[19] Bi H, Feng T, Li B, Han Y (2020). In Vitro and in vivo comparison study of Electrospun PLA and PLA/PVA/SA Fiber membranes for Wound Healing. Polym (Basel).

[20] Hesketh M, Sahin KB, West ZE, Murray RZ (2017). Macrophage phenotypes regulate scar formation and chronic Wound Healing. Int J Mol Sci.

[21] Reel B, Sala-Newby GB, Huang WC, Newby AC (2011). Diverse patterns of cyclooxygenase-independent metalloproteinase gene regulation in human monocytes. Br J Pharmacol.

[22] Garcia-Silva MR, Cabrera-Cabrera F, das Neves RF, Souto-Padrón T, de Souza W, Cayota A (2014). Gene expression changes induced by Trypanosoma cruzi shed microvesicles in mammalian host cells: relevance of tRNA-derived halves. Biomed Res Int.

[23] Tan J, Wu J (2017). Current progress in understanding the molecular pathogenesis of burn scar contracture. Burns Trauma.

[24] Higashi AY, Aronow BJ, Dressler GR (2019). Expression profiling of fibroblasts in chronic and Acute Disease Models reveals novel pathways in kidney fibrosis. J Am Soc Nephrol.

[25] Eves PC, Beck AJ, Shard AG, Mac Neil S (2005). A chemically defined surface for the co-culture of melanocytes and keratinocytes. Biomaterials.

[26] Demidova-Rice TN, Hamblin MR, Herman IM (2012). Acute and impaired wound healing: pathophysiology and current methods for drug delivery, part 1: normal and chronic wounds: biology, causes, and approaches to care. Adv Skin Wound Care.

[27] Vijayan AN, Solaimuthu A, Murali P, Gopi J, Korrapati YMTRAP (2022). Decorin mediated biomimetic PCL-gelatin nanoframework to impede scarring. Int J Biol Macromol.

[28] Ogawa R (2022). The most current algorithms for the treatment and Prevention of Hypertrophic Scars and Keloids: a 2020 update of the Algorithms published 10 years ago. Plast Reconstr Surg.

[29] Nunez JH, Strong AL, Comish P, Hespe GE, Harvey J, Sorkin M, Levi B (2023). A review of laser therapies for the treatment of scarring and vascular anomalies. Adv Wound Care (New Rochelle).

[30] Li-Tsang CW, Feng B, Huang L, Liu X, Shu B, Chan YT, Cheung KK (2015). A histological study on the effect of pressure therapy on the activities of myofibroblasts and keratinocytes in hypertrophic scar tissues after burn. Burns.

[31] Wei Z, Wang M, Hong M, Diao S, Liu A, Huang Y, Yu Q, Peng Z (2016). Icariin exerts estrogen-like activity in ameliorating EAE via mediating estrogen receptor β, modulating HPA function and glucocorticoid receptor expression. Am J Transl Res.

[32] Moore AL, Marshall CD, Barnes LA, Murphy MP, Ransom RC, Longaker MT. Scarless wound healing: transitioning from fetal research to regenerative healing. Wiley Interdiscip Rev Dev Biol. 2018;7(2). 10.1002/wdev.309. 10.1002/wdev.309 PMC6485243 29316315

[33] Mackool RJ, Gittes GK, Longaker MT (1998). Scarless healing. The fetal wound. Clin Plast Surg.

[34] Samuels P, Tan AK (1999). Fetal scarless wound healing. J Otolaryngol.

[35] Leavitt T, Hu MS, Marshall CD, Barnes LA, Lorenz HP, Longaker MT (2016). Scarless wound healing: finding the right cells and signals. Cell Tissue Res.

[36] Pan BH, Zhang Q, Lam CH, Yuen HY, Kuang S, Zhao X (2022). Petite miracles: insight into the nanomanagement of scarless wound healing. Drug Discov Today.

[37] Selig HF, Lumenta DB, Giretzlehner M, Jeschke MG, Upton D, Kamolz LP (2012). The properties of an “ideal” burn wound dressing–what do we need in daily clinical practice? Results of a worldwide online survey among burn care specialists. Burns.

[38] Li XT, Zhang Y, Chen GQ (2008). Nanofibrous polyhydroxyalkanoate matrices as cell growth supporting materials. Biomaterials.

[39] Levin A, Sharma V, Hook L, García-Gareta E (2018). The importance of factorial design in tissue engineering and biomaterials science: optimisation of cell seeding efficiency on dermal scaffolds as a case study. J Tissue Eng.

[40] Weng W, He S, Song H, Li X, Cao L, Hu Y, Cui J, Zhou Q, Peng H, Su J (2018). Aligned Carbon Nanotubes reduce hypertrophic scar via regulating cell behavior. ACS Nano.

[41] Guo X, Liu Y, Bera H, Zhang H, Chen Y, Cun D, Foderà V, Yang M (2020). α-Lactalbumin-based Nanofiber Dressings improve burn Wound Healing and reduce scarring. ACS Appl Mater Interfaces.

[42] Liu C, Zhu Y, Lun X, Sheng H, Yan A (2022). Effects of wound dressing based on the combination of silver@curcumin nanoparticles and electrospun chitosan nanofibers on wound healing. Bioengineered.

[43] Kim HS, Chen J, Wu LP, Wu J, Xiang H, Leong KW, Han J (2020). Prevention of excessive scar formation using nanofibrous meshes made of biodegradable elastomer poly(3-hydroxybutyrate-co-3-hydroxyvalerate). J Tissue Eng.

[44] Ahn S, Chantre CO, Gannon AR, Lind JU, Campbell PH, Grevesse T, O’Connor BB, Parker KK (2018). Soy Protein/Cellulose Nanofiber Scaffolds mimicking skin extracellular matrix for enhanced Wound Healing. Adv Healthc Mater.

[45] Fang Y, Han Y, Wang S, Chen J, Dai K, Xiong Y, Sun B (2022). Three-dimensional printing bilayer membranous nanofiber scaffold for inhibiting scar hyperplasia of skin. Biomater Adv.

[46] Su C, Chen J, Xie X, Gao Z, Guan Z, Mo X, Wang C, Hou G (2022). Functionalized Electrospun double-layer Nanofibrous Scaffold for Wound Healing and Scar Inhibition. ACS Omega.

[47] Jiang Z, Zhao L, He F, Tan H, Li Y, Tang Y, Duan X, Li Y (2021). Palmatine-loaded electrospun poly(ε-caprolactone)/gelatin nanofibrous scaffolds accelerate wound healing and inhibit hypertrophic scar formation in a rabbit ear model. J Biomater Appl.

[48] Wu F, Yuan Z, Shafiq M, Zhang L, Rafique M, Yu F, El-Newehy M, El-Hamshary H, Morsi Y, Xu Y, Mo X (2022). Synergistic effect of glucagon-like peptide-1 analogue liraglutide and ZnO on the antibacterial, hemostatic, and wound healing properties of nanofibrous dressings. J Biosci Bioeng.

[49] Zhang D, Li L, Shan Y, Xiong J, Hu Z, Zhang Y, Gao J (2019). In vivo study of silk fibroin/gelatin electrospun nanofiber dressing loaded with astragaloside IV on the effect of promoting wound healing and relieving scar. J DRUG DELIV SCI TEC.

[50] Kim TH, Jung Y, Kim SH (2018). Nanofibrous Electrospun Heart Decellularized Extracellular Matrix-Based Hybrid Scaffold as Wound Dressing for reducing scarring in Wound Healing. Tissue Eng Part A.

[51] Cheng L, Sun X, Chen L, Zhang L, Wang F, Zhang Y, Pan G, Zhang Y, Zhang L, Cui W (2020). Nano-in-micro electronspun membrane: merging nanocarriers and microfibrous scaffold for long-term scar inhibition. CHEM ENG J.

[52] Guo S, Kang G, Phan DT, Hsu MN, Por YC, Chen CH (2018). Polymerization-Induced phase separation formation of structured hydrogel particles via Microfluidics for Scar therapeutics. Sci Rep.

[53] He J, Meng X, Meng C, Zhao J, Chen Y, Zhang Z, Zhang Y (2022). Layer-by-layer Pirfenidone/Cerium oxide Nanocapsule Dressing promotes Wound Repair and prevents scar formation. Molecules.

[54] Lv Y, Xu Y, Sang X, Li C, Liu Y, Guo Q, Ramakrishna S, Wang C, Hu P, Nanda HS (2022). PLLA-gelatin composite fiber membranes incorporated with functionalized CeNPs as a sustainable wound dressing substitute promoting skin regeneration and scar remodeling. J Mater Chem B.

[55] Pandey VK, Ajmal G, Upadhyay SN, Mishra PK (2020). Nanofibrous scaffold with curcumin for anti-scar wound healing. Int J Pharm.

[56] Singh S, Gupta A, Sharma D, Gupta B (2018). Dextran based herbal nanobiocomposite membranes for scar free wound healing. Int J Biol Macromol.

[57] Ekambaram R, Dharmalingam S (2020). Fabrication and evaluation of electrospun biomimetic sulphonated PEEK nanofibrous scaffold for human skin cell proliferation and wound regeneration potential. Mater Sci Eng C Mater Biol Appl.

[58] Gholipourmalekabadi M, Seifalian AM, Urbanska AM, Omrani MD, Hardy JG, Madjd Z, Hashemi SM, Ghanbarian H, Brouki Milan P, Mozafari M, Reis RL, Kundu SC, Samadikuchaksaraei A (2018). 3D protein-based Bilayer Artificial skin for the guided Scarless Healing of Third-Degree burn wounds in vivo. Biomacromolecules.

[59] Sharifi E, Sadati SA, Yousefiasl S, Sartorius R, Zafari M, Rezakhani L, Alizadeh M, Nazarzadeh Zare E, Omidghaemi S, Ghanavatinejad F, Jami MS, Salahinejad E, Samadian H, Paiva-Santos AC, De Berardinis P, Shafiee A, Tay FR, Pourmotabed S, Makvandi P (2022). Cell loaded hydrogel containing Ag-doped bioactive glass-ceramic nanoparticles as skin substitute: antibacterial properties, immune response, and scarless cutaneous wound regeneration. Bioeng Transl Med.

[60] Kalirajan C, Palanisamy T (2020). Bioengineered Hybrid Collagen Scaffold Tethered with Silver-Catechin Nanocomposite modulates angiogenesis and TGF-β toward Scarless Healing in Chronic Deep Second Degree Infected Burns. Adv Healthc Mater.

[61] Kalirajan C, Palanisamy T (2019). A ZnO-curcumin nanocomposite embedded hybrid collagen scaffold for effective scarless skin regeneration in acute burn injury. J Mater Chem B.

[62] Rahman M, Dutta NK, Roy Choudhury N (2020). Magnesium Alloys with Tunable Interfaces as Bone Implant materials. Front Bioeng Biotechnol.

[63] Pilehvar-Soltanahmadi Y, Akbarzadeh A, Moazzez-Lalaklo N, Zarghami N (2016). An update on clinical applications of electrospun nanofibers for skin bioengineering. Artif Cells Nanomed Biotechnol.

[64] Kumar PS, Sundaramurthy J, Sundarrajan S (2014). Hierarchical electrospun nanofibers for energy harvest ing, production and environmental remediation. Energy Environ Sci.

[65] Wang X, Ding B, Li B (2013). Biomimetic electrospun nanofibrous structures for tissue engineering. Mater Today (Kidlington).

[66] Vijayavenkataraman S (2020). Nerve guide conduits for peripheral nerve injury repair: a review on design, materials and fabrication methods. Acta Biomater.

[67] Barna M, Kucera A, Hladícova M, Kucera M (2007). Der wundheilende Effekt einer Symphytum-Herba-Extrakt-Creme (Symphytum x uplandicum Nyman): Ergebnisse einer randomisierten, kontrollierten doppelblindstudie [Wound healing effects of a Symphytum herb extract cream (Symphytum x uplandicum NYMAN:): results of a randomized, controlled double-blind study]. Wien Med Wochenschr.

[68] Chen H, Lui YS, Tan ZW, Lee JYH, Tan NS, Tan LP (2019). Migration and phenotype control of human dermal fibroblasts by Electrospun Fibrous Substrates. Adv Healthc Mater.

[69] Norzain N, Lin W. Orientated and diameter-controlled fibrous scaffolds fabricated using the centrifugal electrospinning technique for stimulating the behaviors of fibroblast cells J IND TEXT. 2021; 152808372098812.

[70] Chen Y, Shafiq M, Liu M, Morsi Y, Mo X (2020). Advanced fabrication for electrospun three-dimensional nanofiber aerogels and scaffolds. Bioact Mater.

[71] de Aquino AB, Blank AF, Santana LC (2015). Impact of edible chitosan-cassava starch coatings enriched with Lippia gracilis Schauer genotype mixtures on the shelf life of guavas (Psidium guajava L.) during storage at room temperature. Food Chem.

[72] Kim HS, Sun X, Lee JH, Kim HW, Fu X, Leong KW (2019). Advanced drug delivery systems and artificial skin grafts for skin wound healing. Adv Drug Deliv Rev.

[73] Wright JB, Lam K, Burrell RE (1998). Wound management in an era of increasing bacterial antibiotic resistance: a role for topical silver treatment. Am J Infect Control.

[74] Gómez-Estaca J, López de Lacey A, López-Caballero ME, Gómez-Guillén MC, Montero P (2010). Biodegradable gelatin-chitosan films incorporated with essential oils as antimicrobial agents for fish preservation. Food Microbiol.

[75] Kakar MU, Khan K, Akram M, Sami R, Khojah E, Iqbal I, Helal M, Hakeem A, Deng Y, Dai R (2021). Synthesis of bimetallic nanoparticles loaded on to PNIPAM hybrid microgel and their catalytic activity. Sci Rep.

[76] Chen J, Wang H, Mei L, Wang B, Huang Y, Quan G, Lu C, Peng T, Pan X, Wu C (2020). A pirfenidone loaded spray dressing based on lyotropic liquid crystals for deep partial thickness burn treatment: healing promotion and scar prophylaxis. J Mater Chem B.

[77] Huang W, Wang Y, Huang Z, Wang X, Chen L, Zhang Y, Zhang L (2018). On-Demand Dissolvable Self-Healing Hydrogel based on Carboxymethyl Chitosan and Cellulose Nanocrystal for deep partial thickness burn Wound Healing. ACS Appl Mater Interfaces.

[78] Zhang Z, Liu Y, Chen Y, Li L, Lan P, He D, Song J, Zhang Y (2019). Transdermal Delivery of 5-Aminolevulinic acid by Nanoethosome gels for photodynamic therapy of hypertrophic scars. ACS Appl Mater Interfaces.

[79] Cheng H, Shi Z, Yue K, Huang X, Xu Y, Gao C, Yao Z, Zhang YS, Wang J (2021). Sprayable hydrogel dressing accelerates wound healing with combined reactive oxygen species-scavenging and antibacterial abilities. Acta Biomater.

[80] Thammawithan S, Srichaiyapol O, Siritongsuk P, Daduang S, Klaynongsruang S, Prapasarakul N, Patramanon R (2021). Anisotropic Silver Nanoparticles Gel Exhibits Antibacterial Action and reduced scar formation on Wounds contaminated with Methicillin-Resistant Staphylococcus pseudintermedius (MRSP) in a mice Model. Anim (Basel).

[81] Zhang Z, Zhang Y, Li W, Ma L, Wang E, Xing M, Zhou Y, Huan Z, Guo F, Chang J. Curcumin/Fe-SiO2 nano composites with multisynergistic effects for scar inhibition and hair follicle regeneration during burn wound healing. Appl Mater Today. 2021 June;1:23:101065.

[82] Yu Z, Meng X, Zhang S, Wang X, Chen Y, Min P, Zhang Z, Zhang Y (2021). IR-808 loaded nanoethosomes for aggregation-enhanced synergistic transdermal photodynamic/photothermal treatment of hypertrophic scars. Biomater Sci.

[83] Zhao CC, Zhu L, Wu Z, Yang R, Xu N, Liang L (2020). Resveratrol-loaded peptide-hydrogels inhibit scar formation in wound healing through suppressing inflammation. REGEN BIOMATER.

[84] Yu F, Khan AUR, Zheng H, Li X, El-Newehy M, El-Hamshary H, Morsi Y, Li J, Wu J, Mo X (2022). A photocrosslinking antibacterial decellularized matrix hydrogel with nanofiber for cutaneous wound healing. Colloids Surf B Biointerfaces.

[85] Manuja A, Raguvaran R, Kumar B, Kalia A, Tripathi BN (2020). Accelerated healing of full thickness excised skin wound in rabbits using single application of alginate/acacia based nanocomposites of ZnO nanoparticles. Int J Biol Macromol.

[86] Singh A, Kochhar D, Jeevanandham S, Kar C, Bhattacharya R, Shakeel A, Mukherjee M (2020). Emergence of Heptazine-Based Graphitic Carbon Nitride within Hydrogel Nanocomposites for Scarless Healing of burn wounds. ACS Appl Polym Mater.

[87] Bhattacharya D, Tiwari R, Bhatia T, Purohit MP, Pal A, Jagdale P, Mudiam MKR, Chaudhari BP, Shukla Y, Ansari KM, Kumar A, Kumar P, Srivastava V, Gupta KC (2019). Accelerated and scarless wound repair by a multicomponent hydrogel through simultaneous activation of multiple pathways. Drug Deliv Transl Res.

[88] Jin L, Guo X, Gao D, Liu Y, Ni J, Zhang Z, Huang Y, Xu G, Yang Z, Zhang X, Jiang X (2022). An NIR photothermal-responsive hybrid hydrogel for enhanced wound healing. Bioact Mater.

[89] Zhao M, Zhou M, Gao P, Zheng X, Yu W, Wang Z, Li J, Zhang J (2022). AgNPs/nGOx/Apra nanocomposites for synergistic antimicrobial therapy and scarless skin recovery. J Mater Chem B.

[90] Zheng X, Ding Z, Cheng W, Lu Q, Kong X, Zhou X, Lu G, Kaplan DL (2020). Microskin-inspired Injectable MSC-Laden Hydrogels for Scarless Wound Healing with Hair follicles. Adv Healthc Mater.

[91] Liu L, Ding Z, Yang Y, Zhang Z, Lu Q, Kaplan DL (2021). Asiaticoside-laden silk nanofiber hydrogels to regulate inflammation and angiogenesis for scarless skin regeneration. Biomater Sci.

[92] Li M, Liang Y, He J, Healing (2020). CHEM MATER.

[93] Qasemi S, Ghaemy M (2020). Novel superabsorbent biosensor nanohydrogel based on gum tragacanth polysaccharide for optical detection of glucose. Int J Biol Macromol.

[94] Dorati R, Medina JL, DeLuca PP, Leung KP (2018). Development of a topical 48-H release Formulation as an anti-scarring treatment for deep partial-thickness Burns. AAPS PharmSciTech.

[95] Kunkemoeller B, Kyriakides TR (2017). Redox Signaling in Diabetic Wound Healing regulates Extracellular Matrix Deposition. Antioxid Redox Signal.

[96] Bryan N, Ahswin H, Smart N, Bayon Y, Wohlert S, Hunt JA (2012). Reactive oxygen species (ROS)--a family of fate deciding molecules pivotal in constructive inflammation and wound healing. Eur Cell Mater.

[97] Xie P, Dolivo DM, Jia S, Cheng X, Salcido J, Galiano RD, Hong SJ, Mustoe TA (2020). Liposome-encapsulated statins reduce hypertrophic scarring through topical application. Wound Repair Regen.

[98] Jiang K, Chen Y, Zhao D, Cheng J, Mo F, Ji B, Gao C, Zhang C, Song J (2020). A facile and efficient approach for hypertrophic scar therapy via DNA-based transdermal drug delivery. Nanoscale.

[99] Sun J, Zheng Y, Tian D, Li D, Liu Z, Zhang X, Wu Z (2022). A cell membrane repair protein-based nanoformulation with multiple actuators for scarless wound healing. J Mater Chem B.

[100] Chen YY, Lu YH, Ma CH, Tao WW, Zhu JJ, Zhang X (2017). A novel elastic liposome for skin delivery of papain and its application on hypertrophic scar. Biomed Pharmacother.

[101] Yang BY, Zhou ZY, Liu SY, Shi MJ, Liu XJ, Cheng TM, Deng GY, Tian Y, Song J, Li XH (2022). Porous Se@SiO2 nanoparticles enhance Wound Healing by ROS-PI3K/Akt pathway in dermal fibroblasts and reduce scar formation. Front Bioeng Biotechnol.

[102] Weisleder N, Takizawa N, Lin P, Wang X, Cao C, Zhang Y, Tan T, Ferrante C, Zhu H, Chen PJ, Yan R, Sterling M, Zhao X, Hwang M, Takeshima M, Cai C, Cheng H, Takeshima H, Xiao RP, Ma J (2012). Recombinant MG53 protein modulates therapeutic cell membrane repair in treatment of muscular dystrophy. Sci Transl Med.

[103] Jeong Dw, Kim TS, Chung YW, Lee BJ, Kim IY (2002). Selenoprotein W is a glutathione-dependent antioxidant in vivo. FEBS Lett.

[104] Plotczyk M, Jiménez F, Limbu S, Boyle CJ, Ovia J, Almquist BD, Higgins CA (2023). Anagen hair follicles transplanted into mature human scars remodel fibrotic tissue. NPJ Regen Med.

[105] Marshall CD, Hu MS, Leavitt T, Barnes LA, Lorenz HP, Longaker MT (2018). Cutaneous scarring: Basic Science, current treatments, and future directions. Adv Wound Care (New Rochelle).

[106] Thomas S (1990). Wound Management and Dressings.

[107] Baeck M, Marot L, Nicolas JF, Pilette C, Tennstedt D, Goossens A (2009). Allergic hypersensitivity to topical and systemic corticosteroids: a review. Allergy.

[108] Niessen FB, Spauwen PH, Schalkwijk J, Kon M (1999). On the nature of hypertrophic scars and keloids: a review. Plast Reconstr Surg.

[109] Singer AJ, Clark RAF (1999). Mechanisms of disease—cutaneous wound healing. N Engl J Med.

[110] Gan J, Liu C, Li H, Wang S, Wang Z, Kang Z, Huang Z, Zhang J, Wang C, Lv D, Dong L (2019). Accelerated wound healing in diabetes by reprogramming the macrophages with particle-induced clustering of the mannose receptors. Biomaterials.

[111] Wo Y, Zhang Z, Zhang Y, Zhang Z, Wang K, Mao X, Su W, Li K, Cui D, Chen J (2014). Enhanced in vivo delivery of 5-fluorouracil by ethosomal gels in rabbit ear hypertrophic scar model. Int J Mol Sci.

[112] Li Z, Xu K, Qin L, Zhao D, Yang N, Wang D, Yang Y (2023). Hollow nanomaterials in Advanced Drug Delivery Systems: from single- to multiple shells. Adv Mater.

[113] Tang H, Xue Y, Li B, Xu X, Zhang F, Guo J, Li Q, Yuan T, Chen Y, Pan Y, Ping Y, Li D (2022). Membrane-camouflaged supramolecular nanoparticles for codelivery of chemotherapeutic and molecular-targeted drugs with siRNA against patient-derived pancreatic carcinoma. Acta Pharm Sin B.

[114] Ishihara J, Ishihara A, Sasaki K, Lee SS, Williford JM, Yasui M, Abe H, Potin L, Hosseinchi P, Fukunaga K, Raczy MM, Gray LT, Mansurov A, Katsumata K, Fukayama M, Kron SJ, Swartz MA, Hubbell JA (2019). Targeted antibody and cytokine cancer immunotherapies through collagen affinity. Sci Transl Med.

[115] Chen L, Huang Q, Zhao T, Sui L, Wang S, Xiao Z, Nan Y, Ai K (2021). Nanotherapies for sepsis by regulating inflammatory signals and reactive oxygen and nitrogen species: New insight for treating COVID-19. Redox Biol.

[116] Hoshyar N, Gray S, Han H, Bao G (2016). The effect of nanoparticle size on in vivo pharmacokinetics and cellular interaction. Nanomed (Lond).

[117] Tan Y, Suarez A, Garza M, Khan AA, Elisseeff J, Coon D (2020). Human fibroblast-macrophage tissue spheroids demonstrate ratio-dependent fibrotic activity for in vitro fibrogenesis model development. Biomater Sci.

[118] Choi WS, Kim JH, Ahn CB, Lee JH, Kim YJ, Son KH, Lee JW (2021). Development of a Multi-Layer skin substitute using human hair keratinic extract-based hybrid 3D Printing. Polym (Basel).

[119] Wu Z, Hong Y (2019). Combination of the Silver-Ethylene Interaction and 3D Printing to develop Antibacterial Superporous Hydrogels for Wound Management. ACS Appl Mater Interfaces.

[120] Liang J, Zeng H, Qiao L, Jiang H, Ye Q, Wang Z, Liu B, Fan Z (2022). 3D printed Piezoelectric Wound dressing with dual Piezoelectric Response Models for Scar-Prevention Wound Healing. ACS Appl Mater Interfaces.

[121] Wilgus TA, Wulff BC. The importance of mast cells in dermal scarring. Adv Wound Care (New Rochelle). 2014;3(4):356–65. 10.1089/wound.2013.0457 PMC3985512 24757590

